# Groundwater of the Crimean peninsula: a first systematic study using stable isotopes

**DOI:** 10.1080/10256016.2019.1650743

**Published:** 2019-08-16

**Authors:** Yuri V. Dublyansky, Alexander B. Klimchouk, Sergey V. Tokarev, Gennady N. Amelichev, Christoph Spötl

**Affiliations:** a Institute of Geology, Innsbruck University, Innsbruck, Austria; b Institute of Geological Sciences, National Academy of Science of Ukraine, Kiev, Ukraine; c Department of Geography, V. I. Vernadsky Crimean Federal University, Simferopol, Republic of Crimea

**Keywords:** Crimea, groundwater, hydrogen-2, isotope hydrology, oxygen-18, recharge, water–rock interaction

## Abstract

Karst springs in the Main Range of the Crimean Mountains and the Crimean Piedmont show a restricted range of values (δ^18^O = –10.5 to –8.0 ‰, δ^2^H = –72 to –58 ‰), somewhat more negative than the weighted mean of meteoric precipitation. This suggests preferential recharge at higher elevations during winter months. Groundwater tapped by boreholes splits in three groups. A first group has isotopic properties similar to those of the springs. The second group shows significantly lower values (δ^18^O = –13.3 to –12.0 ‰, δ^2^H = –95 to –82 ‰), suggesting recharge during colder Pleistocene times. The third group has high isotope values (δ^18^O = –2.5 to +1.0 ‰, δ^2^H = –24 to –22 ‰); the data points are shifted to the right of the Local Meteoric Water Line, suggesting water–rock exchange processes in the aquifer. These boreholes are located in the Crimean Plains and discharge mineralized (ca. 25 g L^−1^) thermal (65°C) water from a depth of 1600–1800 m. Groundwater associated with mud volcanoes on the Kerch peninsula have distinct isotope characteristics (δ^18^O = –1.6 to +9.4 ‰, δ^2^H = –30 to –18 ‰). Restricted δ^2^H variability along with variable and high δ^18^O values suggest water–rock interactions at temperatures exceeding 95 °C.

## 1. Introduction

Over its known history, the economic development of Crimea has been limited by the availability of water. Around the middle of the twentieth century, the water resources of Crimea totalled 0.83 km^3^, concentrated primarily in the Crimean Mountains. Resources in the Crimean Plains amounted to only 0.04 km^3^, and this part of the peninsula has been largely unsuitable for industrial or agricultural development [1]. Water resources of Crimea have been studied extensively since the 1920s in order to meet the continuously growing demand of domestic and industrial water supply. Hydrogeological surveys and exploratory drilling were performed in all parts of the peninsula between 1940 and the 1970s [2,3]. From 1949 onwards, thousands of boreholes were drilled in the northern part of Crimea, and yearly extraction of water from deep-seated aquifers reached 0.19 km^3^ in 1960 and 0.55 km^3^ in 1975. Massive water extraction produced a plethora of adverse effects, including region-scale depressions in Miocene aquifers, trans-formational flow and contamination, as well as, in coastal areas, ingressions of marine waters into the aquifers [1].

Since 1963, the North Crimean Canal (constructed between 1961 and 1971) started to deliver Dnieper river water from mainland Ukraine to Crimea. The water was primarily used for irrigation and water supply in the northern and eastern parts of the peninsula. The North Crimean Canal had dramatically increased the water resources of the peninsula. Contributing up to ca. 3.8 km^3^ per year, the share of the Dnieper water in the water balance of Crimea in 1963–2014 ranged between 70 and 86 % [4].

In 2014, due to the political confrontation between Ukraine and Russia, the 50 year-long period of ‘water abundance’ ended with the North Crimean Canal being shut off. This resulted in an acute water shortage. Presently, the water demand of ca. 0.8 km^3^ annually must be met by internal resources, but the latter are not sufficient [4]. Groundwater, mostly from karst aquifers, provides ca. 40 % of the water budget in Crimea, and ca. 1.2 million people (ca. 50 % of the Crimean population) depend on karst as the sole source of water. The demand increases dramatically in summer time due to the large number of tourists (about 6 million visitors yearly). In 2018, ca. 0.12 km^3^ of water was extracted by 1204 artesian wells. This exceeds the maximum sustainable withdrawal (0.04 km^3^; [5]). The overuse further aggravates the exhaustion due to uncontrolled exploitation of the most economically viable shallow aquifers in the past. Deeper aquifers (300–500 m) need to be tapped in order to tackle the water shortage.

Solving the pressing issues of sustainable water supply in Crimea requires a thorough understanding of the aquifers. Although major research efforts have been undertaken in Crimea over the last decades, an important source of information, stable water isotopes, has so far only rarely been applied in hydrogeological studies. Except for waters associated with mud volcanoes on the Kerch peninsula, which recently became the focus of several studies [6,7], published information on the isotopic composition of groundwater in Crimea is scarce. Initial measurements of δ^18^O in 10 springs and one borehole, all located within the Main Range of the Crimean Mountains, were reported by Seletsky et al. [8]. Results from 7 karstic springs and one borehole, located on the northern slope of the Crimean Mountains, were reported by Dublyansky et al. [9]. Recently, Kayukova [10] published isotope data for 26 springs in the Crimean Mountains, and Amelichev et al. [11] reported stable isotope data for one mineral water occurrence on the Crimean Plains.

In this study, we attempt a first systematic stable isotope characterization of the different types of groundwater on the 27,000 km² territories of the Crimean peninsula. We sampled 41 springs, 30 boreholes, 4 wells and 7 mud volcanoes located in different hydrogeological districts. In order to assess the temporal variability of the isotope composition, several sites were sampled over a longer interval; for three karst springs, we obtained time series of different length. Given the size of the study area, its hydrogeological complexity, the range of recharge conditions, and the multitude of aquifers tapped by water supply wells, this study is necessarily a reconnaissance one.

## 2. Geology and hydrogeology of Crimea

### 2.1. Geography and geology

**Figure 1.**
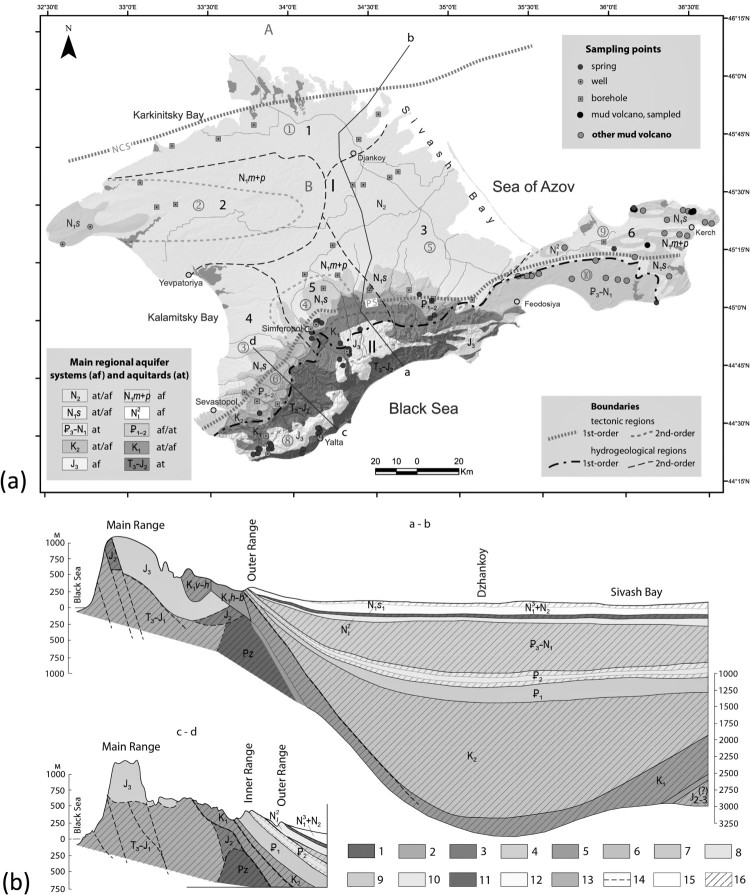
Tectonic and hydrogeological overview of Crimea. (a): Tectonic regions (after Yudin [12]; capitalized letters): 1st-order regions: A – Ukrainian Plate, B – Scythian Plate, C – Crimean Mountains foldthrust region. Boundaries between 1st-order regions: NCS – North-Crimean suture, PS – Piedmont suture. 2nd-order regions (circled numerals): 1 – Karkinitsky depression, 2 – Tarkhankutsky-Novoselovsky uplift, 3 – Alminskaya depression, 4 – Simferopol uplift, 5 – Indolo-Kubansky depression, 6 – Cuesta homocline, 7 – Piedmont structural zone, 8 – Mountain structural zone, 8a – Baydarsky depression, 9 – North-Kerch retrothrust zone, 10 – South-Kerch thrust zone. Hydrogeological regions: 1st-order regions (after Shestopalov et al. [13]; Roman numerals): I – Prichernomorsky groundwater system (artesian basin), II – Crimean Mountains groundwater system. 2nd order regions in the Crimean Plains and the Kerch peninsula (after Lushchik et al. [15]; black numerals): 1 – North-Sivash, 2 – Novoselovsky; 3 – Belogorsky; 4 – Alminsky; 5 – Simferopolsky, 6 – Kerch system of small artesian basins. Lines a–b and c–d correspond to hydrogeological cross-sections shown in (b). (b): Simplified hydrogeological cross-sections, approximately corresponding to lines a-b and c-d in (a). Modified from Barabanov et al. [3]. 1 – Palaeozoic metamorphosed rocks (aquitards); 2 – Triassic / Lower Jurassic shales of the Tavricheskaya series (aquitards); 3 – Middle Jurassic sandstones, shales and conglomerates (aquitards); 4 – Upper Jurassic limestones (karst aquifers); 5 – Lower Cretaceous sandstones, conglomerates and limestones (aquitards, locally aquifers); 6 – Upper Cretaceous limestones and marls (locally fractured/karst aquifers), with marls and sandstones at the base (aquitards); 7 – Palaeocene limestones and marls (fractured/karst aquifers); 8 – Eocene limestones and marls (fractured/karst aquifers), with shales at the base (aquitards); 9 – Oligocene clays of the Maikop series (aquitards); 10 – Middle Miocene sands and limestones (aquifers); 11 – Lower-Middle Sarmatian clays (aquitards); 12 – Upper Miocene – Pliocene limestones and sands (aquifers), with clays in upper part (aquitards); 13 – sandy mudstones, presumably of the Middle to Upper Jurassic (locally fractured aquifers); 14 – fault lines; 15 – predominantly aquifer formations; 16 – predominantly aquitard formations. Vertical exaggeration ca. 17x.

The Crimean peninsula comprises two main physiographic provinces, the Crimean Plains in the northern part and the Crimean Mountains in the south (see Supplementary Material, Figure 1). The Crimean Mountains comprise (from south to north) the Main, the Inner, and the Outer Ranges; the latter two are also known as the Crimean Piedmont (Predgorje). The physiographic provinces of the Crimean Plains and the Crimean Mountains correspond to two regional tectonic structures: the epi-Hercynian Scythian Plate and the Alpine Crimean Mountains fold-and-thrust structure (Figure 1(a)). These structures are separated by a regional deep-seated fault, which has also been interpreted as a collision suture (Piedmont Suture; [12]). This low-angle north-dipping thrust fault was active from the early Jurassic until the early Cretaceous. The lower tectonostratigraphic story in the Crimean Mountains is composed mainly of Triassic shale-dominated flysch deposits and Upper Jurassic and Lower Cretaceous limestones. The limestones form rigid massifs interpreted as olistoliths [12]. The upper tectonostratigraphic story comprises alternating shales/marls and carbonate rocks of Upper Cretaceous, Palaeogene, and Neogene age, which form a homocline dipping 5 °–15 ° to the northwest and north.

From the Late Cretaceous to the Palaeogene, the northern periphery of the fold-and-thrust belt of the Crimean Mountains was buried by marine sediments, which were even more abundant further to the north in the Scythian Plate (the Crimean Plains). The Upper Jurassic limestone massifs that now form most of the Main Range were stripped off their Cretaceous–Palaeogene cover during the Neogene–Quaternary uplift. Ensuing erosion separated the Main and the Inner Ranges. The geological and hydraulic continuity of the homocline was completely breached in the northwestern sectors, but was preserved in the central and eastern parts of the mountain region.

The Inner and the Outer ranges constituting the Crimean Piedmont rim the Crimean Mountains from the northwest and north, forming a 130 km-long arch. Cuestas of the Inner Range are more prominent and are built up of Upper Cretaceous, Palaeocene and Eocene carbonate strata. Further to the northwest and north, Cretaceous to Palaeocene strata have a steeper dip and plunge to great depth. Cuestas of the Outer Range are built up of Neogene carbonates. The thickness of the Neogene strata increases northward, reaching several hundred metres in the subsurface of the Crimean Plains. The basement of the Scythian Plate is composed of strongly deformed Palaeozoic rocks dominated by metamorphosed shales and carbonates.

### 2.2. Hydrogeology

Two main hydrogeological domains are distinguished within the Crimean peninsula (Figure 1(a)), the Crimean Mountains groundwater system and the southern part of the Prichernomorsky groundwater system (artesian basin) which encompasses the Crimean Plains and the Piedmont [13]. Structural depressions in the basement of the Scythian Plate within the Crimean part of the Prichernomorsky system form second-order artesian basins (North-Sivash, Belogorsky and Alminsky). The Kerch peninsula hosts a suite of small-scale artesian basins. Schematic hydrogeological cross-sections are presented in Figure 1(b).

#### 2.2.1. Crimean Mountains groundwater system

The main groundwater resources in the Crimean Mountains are associated with the karstified Upper Jurassic limestones, which reach a thickness of up to 1 km. Terrigenous and terrigenous–volcanogenic deposits form the basement of the karstified plateaus (yailas) in the Main Range and show low hydraulic conductivities. The plateaus serve as the main recharge areas. Limestone massifs of the Main Range provide relatively small storage of karst water in the phreatic zone, which discharges via more than 2000 springs located on the periphery of the plateaus, mostly within the 200–600 m a.s.l. altitudinal interval. Discharge of the karst springs ranges from a few to hundreds of litres per second [3] with 19 springs having discharges exceeding 100 L s^−1^ (and accounting for ca. 75 % of the total discharge). This indicates that the groundwater flow is focused into a few highly transmissive karst conduits.

Where Upper Jurassic limestones plunge underneath the Cretaceous cover outside the massifs they form confined aquifers providing additional recharge for the adjacent Prichernomorsky system. For example, waters recharging in the Dolgorukovskaya and Karabi yailas feed the aquifers of the Prichernomorsky system in the Simferopol uplift and the Belogorsky basin [3,14].

Groundwater in the Upper Jurassic limestones, even when confined (e.g. in the Baydarsky depression), belongs to the zone of active water exchange and shows 0.3–0.5 g L^−1^ total dissolved solids (TDS). Some boreholes located outside the massifs at intersections of tectonic faults tap confined Na-Cl-type (e.g. Krasnaya cave area) or Na-HCO_3_-type (e.g. Chernye Vody area) waters exceeding 3 g L^−1^. These waters are commonly enriched in He, Rn, H_2_S, CH_4_, and higher hydrocarbons, suggesting admixture of deep-seated fluids. High TDS, high contents of dissolved gases (CO_2_ and H_2_S) and minor elements were also reported from some springs in the eastern part of the Crimea Mountains associated with faults [3,14,15].

#### 2.2.2. Southern part of the Prichernomorsky system

The Prichernomorsky system extends as far south as the Crimean Piedmont, where Upper Cretaceous, Palaeogene and Neogene strata are uplifted, tilted toward the north-northwest and exposed within the Inner and the Outer Ranges and on the northern slope of the Crimean Mountains. This is the recharge area of the groundwater system located underneath the Crimean Plains.

The upper aquifers of the system are unconfined and the rocks crop out in the Southern Longitudinal depression in the Piedmont as well as in the Tarkhankut and the Novoselovsky uplifts. The aquifers are recharged by infiltration of meteoric precipitation and, partly, by sub-river channel flow on the northern slope of the Main Range and in the Piedmont, where river valleys incise into Cretaceous, Palaeogene and Neogene strata. As the groundwater flows deeper towards the Crimean Plains it acquires significant hydraulic head. The pressure difference between the recharge area on the Piedmont and the submerged areas may exceed 30 bar in the Neogene aquifers and 100 bar in Upper Cretaceous aquifers [3,15]. The Aptian-Albian and some Upper Cretaceous strata, the thick Oligocene-Lower Miocene Maikop series deposits, as well as thin but continuous beds of Lower Sarmatian clays act as aquitards [3,14–16]. Aquifers underlying the Crimean plains are shown in Figure 1(b); their description is provided in Supplementary Materials.

## 3. Methods

**Figure 2.**
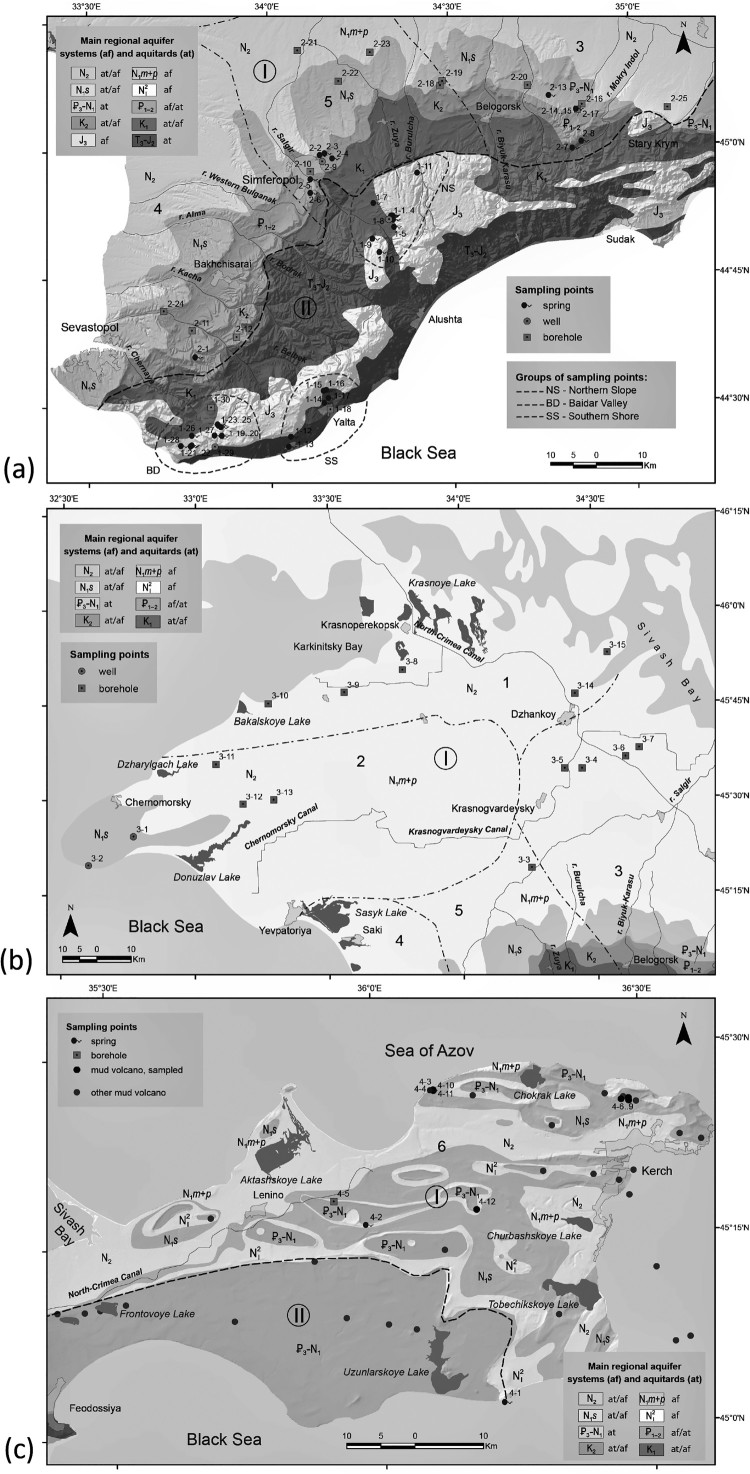
Geological maps of the studied regions of Crimea showing locations of samples and boundaries of hydrogeological units (cf. Figure 1). (a): Main Range of the Crimean Mountains and the Crimean Piedmont; (b): the Crimean Plains; (c): the Kerch peninsula.

We sampled 41 natural springs, 4 draw-wells and 30 boreholes in different parts of Crimea, as well as water from 7 mud volcanoes on the Kerch peninsula (Figure 2). Samples were collected in 1.5 mL vials with screw caps and PTFE/Red rubber septa and stored at +5 °C until shipment to the laboratory. All isotope analyses were performed at the Institute of Geology, Innsbruck University, Austria. Samples collected between 2009 and 2014 were analysed using isotope ratio mass spectrometry (IRMS). From mid-2014 onward, analyses were performed by isotope ratio infrared spectroscopy (IRIS).

*IRMS* — The isotopic composition of hydrogen was measured using a Delta V Advantage mass spectrometer, equipped with an AS3000 autosampler, a thermal-combustion/elemental analyzer (TC/EA) unit, and a ConFlo III interface (all Thermo Fisher Scientific). The TC/EA unit was converted to ‘reverse gas flow’ configuration to improve precision [17]. Each sample was characterized by 9 injections of 0.4 μL each. To check the reproducibility of the results, 10 % of the samples were randomly re-measured. The precision of the δ^2^H measurements was 1 ‰ (1σ). The isotopic composition of oxygen was measured on a Delta Plus XL IRMS linked to a GasBench II (all Thermo Fisher Scientific) using the CO_2_ equilibration technique [18]. The precision of these measurements was 0.1 ‰ (1σ).

*IRIS* — The IRIS method allows direct measurement of water vapour with almost no sample preparation. We used the L2140i analyzer equipped with the A0211 high-precision vaporizer (Picarro, USA). The analytical protocol and the data processing designed to minimize memory effects were adopted from [19]. The precision of the measurements was better than 1 ‰ for δ^2^H and 0.1 ‰ for δ^18^O (1σ).

The measured δ^2^H and δ^18^O values were normalized to the VSMOW scale by using laboratory reference waters, calibrated against IAEA primary standards (VSMOW2, SLAP2, and GISP). In order to ensure that the transition from IRMS to IRIS in 2014 did not compromise the integrity of the dataset, several tens of archived samples originally analysed by IRMS were re-measured using IRIS. No significant differences were observed.

## 4. Results

### 4.1. Overview

Most of the data plot within the ±1 ‰ δ^18^O-band of the Crimean Local Meteoric Water Line (LMWL; δ^2^H = 7.3 δ^18^O + 4.8 ‰; *r*^2^ = 0.97, as defined in Dublyansky et al. [20]). Only data from a borehole located in the northernmost part of the Crimean Plains and all but one samples from the Kerch peninsula plot to the right of the LMWL.

### 4.2. Main Ridge of the Crimean Mountains

Samples from the Main Range of the Crimean Mountains can be subdivided into three groups (Figure 2(a)). The *Southern Shore* group comprises 6 springs in the area of Greater Yalta, one of which has elevated H_2_S contents. The *Northern Slope* group comprises 16 karst springs located on the northern slope of the Karabi yaila, the western slope of the Dolgorukovskaya yaila (including the underground river of the Kransaya cave), and at the northern foothills of the Chatyr-Dag plateau, as well as one borehole tapping water in Upper Jurassic rocks on the western foothills of Dolgorukovskaya yaila. The *Baydarsky depression* group comprises 10 karst springs, one draw-well and one borehole, all discharging from Upper Jurassic carbonate rocks.

**Figure 3.**
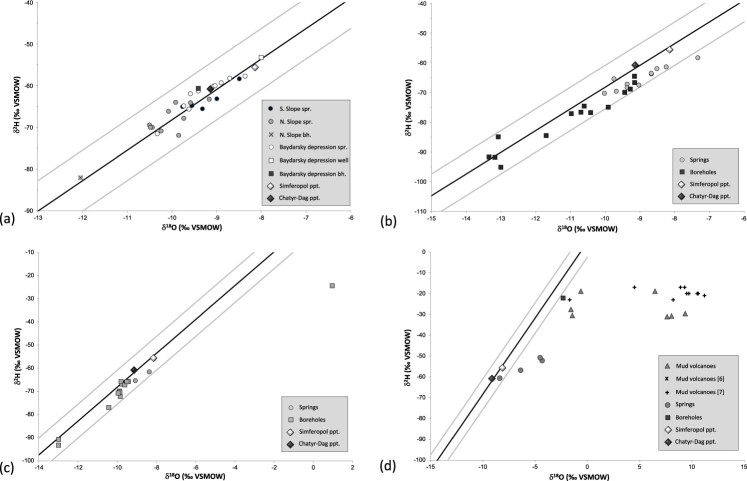
Stable isotope properties of the Crimean groundwater: (a): the Main Range of the Crimean Mountains; (b): the Crimean Piedmont; (c): the Crimean Plains; (d): the Kerch peninsula (isotope data reported by Aydarkozhina and Lavrushin [6] and by Ershov and Levin [7] are also shown). Data are plotted with respect to the Crimean LMWL (black line; Dublyansky et al. [20]) and the ±1 ‰ δ^18^O band (grey lines). Large diamonds are weighted mean values measured at two precipitation-sampling stations, Simferopol and Chatyr-Dag, in 2009–2011.

The water isotope data from the Main Ridge are consistent with the Crimean LMWL (Figure 3(a)). Springs from the Northern Slope of the range have, overall, slightly more depleted values compared to springs from the Southern Shore and the Baydarsky depression.

**Figure 4.**
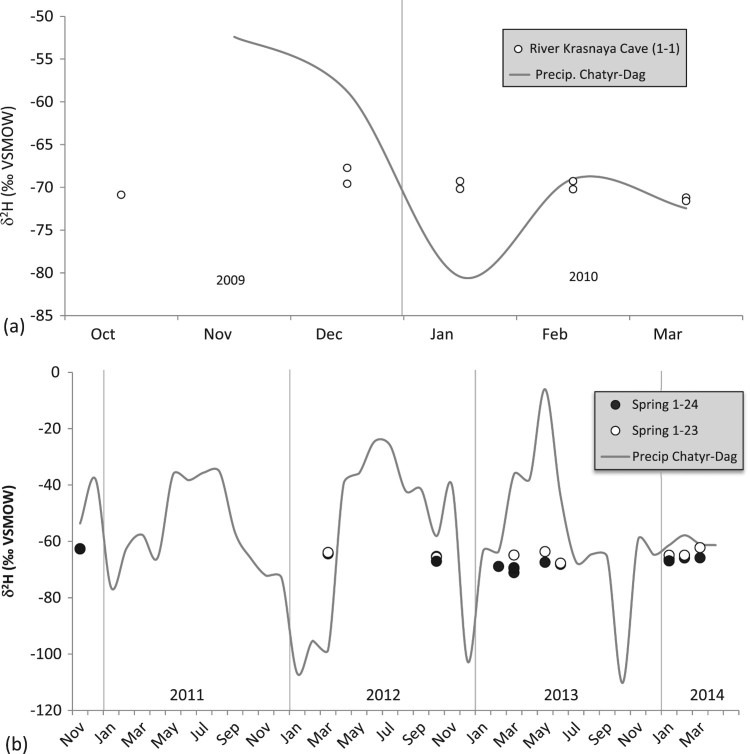
Hydrogen isotope values of water from the underground river in Krasnaya cave (1-1) on Dolgorukovskaya yaila (a), and springs Ogni Grifona (1-23) and Skelsky (1-24) in the Baydarsky depression (b) compared to precipitation on Chatyr-Dag (Dublyansky et al. [20], solid line)

The spring waters are isotopically more depleted than the weighted annual mean of precipitation in Simferopol, reflecting recharge at higher altitudes of the Main Range. The comparison with annual averages of the Chatyr-Dag station is less clear as the latter exhibits larger year-to-year variations [20]. Repeated measurements of the underground river in the Krasnaya cave and at two karst springs in the Baydarsky depression (1-23 and 1-24; here and further in the text site numbers as per Figure 2) show that the isotopic composition of karst water is fairly stable. In the cold season of 2009–2010 the underground river in the Krasnaya cave (1-1) had δ^18^O and δ^2^H values of –10.4 ± 0.3 ‰ and –70.0 ± 1.5 ‰, respectively (Figure 4(a); there might be a subdued maximum in December, but its amplitude is close to the analytical uncertainty). This period is characterized by snowmelt and rain-related flooding, causing high-amplitude fluctuations of the discharge [21]. These changes, however, do not seem to affect the isotopic composition of the water, nor are seasonal variations in the isotopic composition of precipitation reflected in the composition of the karst water. A similar behaviour was documented in time series from two springs in the Baydarsky depression (Figure 4(b)). The springs exhibit similar isotopic compositions that neither respond to drastic swings in compositions of precipitation nor to seasonally variable spring discharge.

Water from a borehole in the Baydarsky depression (1-30) yielded isotope values similar to the springs. In contrast, the monitoring borehole near the Krasnaya cave (1-8) yielded strikingly low isotope values (δ^18^O = –12.9 ± 0.2 ‰; δ^2^H = –83.0 ± 1.0 ‰), lower than the most negative winter precipitation measured in Crimea [20]. This borehole taps deep-seated water from a zone of restricted circulation in a submerged block of Upper Jurassic limestone.

### 4.3. Crimean Piedmont

Natural springs are less common in the Crimean Piedmont than in the Crimean Mountains. Eleven springs, one draw-well, and 13 boreholes were sampled in the Crimean Piedmont (Figure 2(a)). Generally, springs have isotopic compositions similar to their counterparts in the Main Range, just slightly shifted toward higher values (Figure 3(b)). In contrast, boreholes tap waters that are slightly more negative than both spring water in the Piedmont and borehole water in the Crimean Mountains, with four boreholes (2-11, 2-22, 2-23, and 2-24) yielding significantly depleted values (δ^18^O = –13.5 to –13.0 ‰; δ^2^H = –95 to –85 ‰).

### 4.4. Crimean Plains

Natural springs in the Crimean Plains are very rare. For this study 2 draw-wells and 13 boreholes were sampled (Figure 2(b)). All but one data points plot along the Crimean LMWL (Figure 3(c)). Values from draw-wells are isotopically slightly less negative than most of the borehole waters. Tapped water from two boreholes (3-3 and 3-4) is strongly depleted (δ^18^O = –13 ‰, δ^2^H = –95 to –90 ‰). One borehole (3-15) is distinct, because of the isotopically enriched character of its water, which is displaced to the right of the LMWL (δ^18^O = +1 ‰, δ^2^H = –24.5 ‰).

### 4.5. Kerch peninsula

Four springs, one borehole and seven mud volcanoes, some of them with oil shows, were sampled on the Kerch peninsula (Figure 2(c)). Waters on the peninsula are quite different compared to other parts of Crimea in that most of them do not plot on the Crimean LMWL. Only one spring, Kyrk-Chokrak on the Opuk hill (4-1), yielded isotope values consistent with a meteoric provenance (Figure 3(d)). Isotope values of the three other springs are displaced to the right of LMWL, defining a line with a slope of 2.3.

Water from a borehole (4-5) has high isotope values, slightly shifted to the right of the LMWL (δ^18^O = –2.3 ‰, δ^2^H = –22.2 ‰). Waters sampled at mud volcano cones have high ^2^H concentration (δ^2^H > –31 ‰) and are disproportionately enriched in ^18^O, which places the data points to the right of the LMWL (up to δ^18^O = +9.4 ‰).

## 5. Discussion

### 5.1. Springs in the Crimean Mountains and the Piedmont

**Figure 5.**
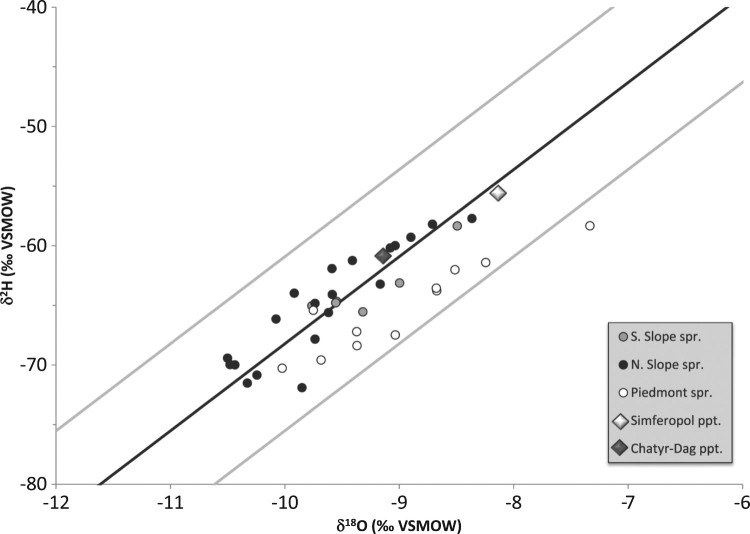
Stable isotope properties of spring waters in the Main Range of the Crimean Mountains and the Crimean Piedmont.

Thirty-seven springs were sampled in the Main Range of the Crimean Mountains and the Piedmont. The isotopic compositions of spring waters are consistent with the LMWL and, with one exception, plot within a ±1 ‰ (δ^18^O) band of this line (Figure 5). The only spring deviating from this line by more than 1 ‰ (2-15) is likely affected by evaporation.

The Crimean spring waters have generally similar characteristics. Although springs from the northern slope of the Main Range have somewhat lighter isotope values, the difference is not significant. All spring waters show lower isotope values than the annual mean of meteoric precipitation in Simferopol. Most of them are also lower than precipitation on the Chatyr-Dag plateau; the latter, however, is variable on an annual basis. The isotopically ‘light’ character of spring water can be explained by (a) predominant winter recharge, and (b) recharge at higher elevations. The mechanisms are likely complementary, but their contributions vary for different springs. For example, high-elevation recharge cannot be invoked for the epikarst spring in the Piedmont (e.g. 2-1). On the other hand, attributing the shift towards lower isotope values to cold-season recharge may also be a simplification, because the difference between cold and warm seasons can be highly variable in this region [20]. This issue is further discussed in Section 5.3.

### 5.2. Karst springs time-series

As discussed in Section 4.2, time series of three sites on the northern slope of the Main Range of the Crimea Mountains, the underground river in the Krasnaya cave (Dolgorukovskaya yaila; 1-1) and two karst springs in the Baydarsky depression (1-23 and 1-24) show stable isotopic compositions that are time-independent (Figure 4). This invariant isotopic composition of water, unaffected neither by discharge rate nor by the composition of atmospheric precipitation in the recharge area, is consistent with the results from karst regions elsewhere, indicating that variations in the isotopic compositions of precipitation are largely smoothed out during the movement of the water through the soil zone and the epikarst [23,24]. The base-flow of karst springs commonly shows isotope values similar to the mean annual composition of precipitation in the recharge area, whereas slight deviations from this value may be caused by major precipitation and resulting flood events [23,24]. The isotopic composition of the springs in the Main Range of the Crimean Mountains is biased toward winter, rather than reflecting the value of the mean annual precipitation (see below).

**Figure 6.**
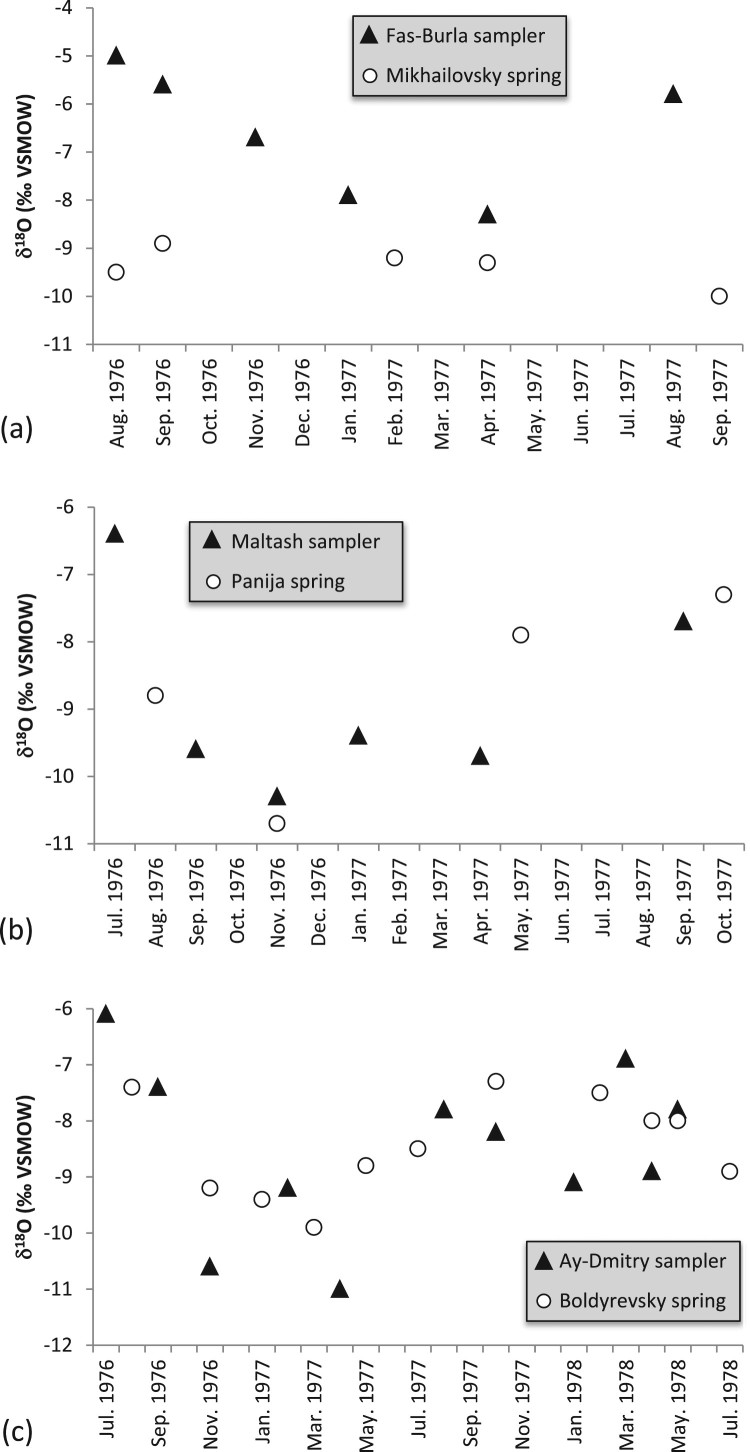
Isotope properties of spring water and local precipitation reported by Seletsky et al. [8]. (a): Panija spring and Mal-Tash precipitation sampler; (b): Boldyrevskiy spring and Ay-Dmitry sampler; (c): Mikhailovsky spring and Fas-Burla sampler.

Previous publications, however, suggest that such stable pattern may not be the only characteristic of the karst springs in Crimea. Seletsky et al. [8] reported δ^18^O time series for four springs along with matching (albeit discontinuous) values of meteoric precipitation measured at adjacent weather stations. Two springs, Panija and Boldyrevsky (located at 560 and 650 m a.s.l., respectively, on the North Slope of the Crimean Mountains) show δ^18^O variations broadly synchronous with variations in meteoric precipitation, albeit with a somewhat reduced amplitude (2.5 and 3.4 ‰ vs. 3.9 and 4.9 ‰ in precipitation, respectively; Figure 6). In contrast, Mikhailovsky spring, located at ca. 300 m a.s.l. on the Southern Slope, shows significantly less variable δ^18^O values (1.1 ‰ range) independent of seasonal variations in precipitation. Both springs show isotopic properties controlled by precipitation discharge water from Upper Jurassic limestone of the Yaltinskaya and the Ay-Petrinskaya yailas. Both are karst springs, but of rather different hydrological characteristics: Panija is the fourth largest karst spring in the Crimean Mountains (discharge 12–8600 L s^−1^; mean 396 L s^−1^), while Boldyrevsky spring is fairly small (discharge 0–100 L s^−1^; mean 12 L s^−1^). Mikailovsky spring (discharge 1–54 L s^−1^; mean 7.5 L s^−1^) discharges in unconsolidated sediments of the Tavricheskaya Series (T_3_-J_2_). Its catchment probably includes parts of the Yaltinskaya and the Ay-Petrinskaya yailas.

The variable response of individual springs to the seasonal isotopic cycle of meteoric precipitation suggests a variable relationship between focused input of precipitation that largely bypasses the epikarst and diffuse input (vadose seepage and vadose flow) largely homogenized within the epikarst [22]. Longer time series from different springs are needed to further address this aspect of karst spring hydrology in Crimea.

### 5.3. Winter recharge of karst springs

The overall isotopically depleted character of spring waters in the Crimean Mountains compared to the local meteoric precipitation is apparent in Figures 3(a) and Figure 4(a). Similarly, Seletsky et al. [8] analysed 10 springs from the southwestern segment of the Main Range of the Crimea Mountains and noted that all springs have δ^18^O values more negative than those of precipitation at the respective altitudes. These authors concluded that (a) the recharge area for springs on both slopes of the mountains are the plateaus of the Main Range, and (b) all springs are fed primarily by winter precipitation. Similarly, δ^18^O and δ^2^H values of several karst springs as well as the underground river in the Krasnaya cave on the northern slope of the Crimean Mountains are isotopically depleted with respect to the annual meteoric precipitation in the respective recharge areas [9]. This observation supports the concept of the predominantly winter recharge of these springs.

As the mean relative humidity on the karst plateaus of the Crimea Mountains in summer is ca. 58 %, only rainfall events exceeding 20 mm d^−1^ will infiltrate, whereas less intense rainfall will evaporate [25]. According to two decades of observations reported by Dublyansky et al. [21], 56–90 % of the precipitation on the Dolgorukovskaya yaila is lost by evaporation between May and October. Based on our time series of monthly samples of precipitation on the Chatyr-Dag plateau (980 m a.s.l., 2011–2013; [20]), we calculated the mean isotopic composition of recharge water, assuming that starting from a certain mean temperature, the water will be removed by evaporation and will not contribute to the recharge. Removing data for those months for which the mean temperature exceeded 15 °C (depending on the year, such periods may be longer or shorter, lasting from May through September or from June through August) yields recharge water with δ^2^H values that is some 10 ‰ more negative than the weighted average of the annual recharge. Although this model is admittedly simplistic (intense precipitation events can contribute to recharge even during the high temperature and low relative humidity periods), it provides a plausible explanation of the depleted character of karst spring water in the Crimean Mountains.

To summarize, winter conditions on the yailas, characterized by low temperatures, relative humidity of ca. 90 %, and multiple events of accumulation and thawing of the snow cover [21], are most favourable for karst water recharge in the Crimean Mountains.

### 5.4. Groundwater from boreholes

#### 5.4.1. ‘Common’ waters

Isotope values of most groundwater samples from the Main Range of the Crimean Mountains, the Piedmont and the Crimean Plains plot within the ±1 ‰ (δ^18^O) band of the LMWL and their δ^18^O and δ^2^H values range between −11 and −8 ‰ and −80 and −55 ‰, respectively. In the Crimean Piedmont, such values are common in boreholes sampling aquifers in Palaeocene–Eocene, Lower Cretaceous and Upper Jurassic units, down to 745 m depth. In the Crimean Plains, this range of values is characteristic of boreholes tapping water from shallow (3–80 m deep) aquifers in Sarmatian–Pontian strata.

#### 5.4.2. Isotopically light groundwater

Water from eight boreholes yielded distinctly low δ^18^O values of −13.3 to −12.0 ‰ and also low δ^2^H values of −95 to −82 ‰. One of these boreholes is located on the northern slope of the Crimean Mountains (1-8), five in the Crimean Piedmont (2-10, 2-11, 2-22, 2-23, and 2-24) and two in the Crimean Plains (3-3 and 3-4).

Borehole 1-8 near the Krasnaya cave has a depth of 60 m and penetrates a downthrown block of Upper Jurassic limestone. The water tapped by this borehole has a relatively low temperature (12 °C), and its chemical and dissolved gas composition is distinct from other karst waters of the Dolgorukovskaya yaila. The water is of Na-HCO_3_-type with TDS values exceeding 1.1 g L^−1^. Dissolved gases comprise CH_4_, H_2_S, He, and ^222^Rn [21], suggesting a significant contribution from a deep-seated groundwater source.

The five boreholes showing low isotopic values in the Piedmont tap freshwater (0.38–0.55 g L^−1^ TDS) of slightly elevated temperatures (25–40 °C). Despite their locations in different hydrogeological districts of the Piedmont (Alminsky for 2-11 and 2-24, and Simferopolsky for 2-10, 2-22 and -23), these boreholes tap Lower Cretaceous, Palaeocene and Eocene aquifers in a homocline which plunges northward underneath younger basinal deposits of the Crimean Plains. The depleted isotope values of groundwater in shallower Palaeocene–Eocene aquifers may be related to localized cross-flow from deeper Lower Cretaceous formations.

In the Crimean Plains, one borehole with lower isotope values taps a Lower Cretaceous aquifer at a depth of 975–1182 m (3-3) and discharges hot water (53 °C). The second borehole (3-4) probably taps the shallower (90–120 m) Sarmatian–Maeotian strata and shows a temperature of 19 °C. The difference to other boreholes tapping the Sarmatian–Maeotian aquifers can be explained by locally ascending cross-formational water sourced from the deep-seated Lower Cretaceous aquifer. Such cross-formational flow may occur in places where the aquitard separating aquifers is breached by some natural process (fault, hypogene karst); it may also occur within the borehole.

Such highly negative values are rarely observed in modern precipitation samples, even during the coldest winter months. Modern precipitation is thus unlikely to be the main source of these waters. A possible explanation involves pre-Holocene recharge of this deep-seated aquifer, reflecting the colder Pleistocene climate characterized by strongly depleted isotope ratios [9].

#### 5.4.3. Isotopically heavy water

Borehole 3-15 near Medvedevka village in the northernmost part of the Crimean Plains is one of the deepest boreholes sampled in this study. It taps the Lower Cretaceous aquifer at a depth of 1600–1800 m. The water is brackish (25.3 g L^−1^) and thermal (65°C at the borehole orifice). The deep-seated water tapped by this borehole has no local recharge. It shows high δ^18^O (+1 ‰) and δ^2^H (−24.5 ‰) values. Similar values were obtained from another borehole and from waters of mud volcanoes on the Kerch peninsula (see below). Apparently, borehole 3-15 taps deep-seated water that underwent significant water–rock isotopic exchange.

### 5.5. Groundwater of the Kerch peninsula

#### 5.5.1. Springs

The Kyrk-Chokrak spring on the Opuk hill (4-1) is the only spring sampled in this study on the Kerch peninsula, which shows an isotopic composition consistent with meteoric precipitation. This is expected as the Kyrk-Chokrak is an epikarst spring, where up to 23 % of its recharge is provided by condensation within the epikarst [26]. The isotopic compositions of the three other springs (4-2, 4-3, and 4-4) are displaced to the right of the LMWL, defining a line with the slope of 2.3. This slope is too small to be attributed to evaporation (the latter is characterized by slopes ranging between 3.9 and 6.8 at relative humidities ranging between 0 and 95 %) [27]. Most likely this line represents a mixing trend between precipitation water and deep-seated groundwater, similar to that sampled in mud volcanoes (see below). This interpretation is supported by the elevated H_2_S content of two of these springs, their moderately high TDS values (14.2–14.3 g L^−1^) and slightly elevated temperatures (15–16 °C, compared to the mean annual temperature in Kerch of ca. 11 °C). It may be inferred, therefore, that deep-seated groundwater provides partial recharge for shallow aquifers feeding the studied springs on the Kerch peninsula.

#### 5.5.2. Mud volcanoes

Kerch peninsula hosts more than 20 mud volcanoes, some of which are presently inactive [28]. Water discharging from volcano mounds are slightly alkaline, has a variable chemical composition (HCO_3_-Cl-Na, Cl-HCO_3_-Na, SO_4_-Cl-Na and Cl-Na-type) and TDS (8.6–23.0 g L^−1^). It contains dissolved CH_4_ (93–98 vol. %), ethane, butane and propane (up to 3.4 vol. %), and small amounts of CO_2_ (0.3 vol. %) and N_2_ (0.1–2.6 vol. %) [29].

Recently Ershov and Levin [7] reported water data from 10 mud volcanoes on the Kerch peninsula. They showed TDS values of 13.2–17.6 g L^−1^ and the Mg-Li geothermometer suggests temperatures at the volcano ‘roots’ of 70–107 °C. These waters show enriched δ^2^H values, ranging from −17 to −21 ‰. In contrast, δ^18^O values show a higher degree of variability (−1.7 to +11.2 ‰) with all data points displaced to the right of the LMWL (Figure 3(d)). Ershov and Levin [7] attributed these compositions to either the deep-seated nature of the ‘roots’ of these mud volcanoes or to water–carbonate rock exchange at the temperature of about 150 °C. A similar explanation of high δ^18^O values (up to +14.2 ‰) in waters from mud volcanoes on the Taman peninsula (east of the Kerch peninsula, across the Kerch strait) was proposed by Buyakaite et al. [30].

Although obtained on different mud volcanoes, results of the present study are consistent with the data of Ershov and Levin [7]. Our data show a somewhat larger δ^2^H range (−30 to −18 ‰) but nearly identical δ^18^O values, ranging from −1.6 to +9.4 ‰ (Figure 3(d)). Water from one borehole (T = 18 °C) in the area has similarly high δ^2^H values and is slightly enriched in ^18^O (Figure 3(d)). Strong and variable enrichment in ^18^O combined with almost invariant δ^2^H values suggests that these waters have experienced variable degrees of water–rock isotope exchange at elevated temperatures. Equilibrium calculations show that in order to obtain δ^18^O_water_ values as high as +12 ‰ via isotopic exchange with limestone (assuming δ^18^O_limestone_ = 0 ‰ VPDB), a minimum temperature of 95 °C is required. This temperature was calculated assuming an infinitely low water/rock ratio; higher temperatures are needed if the water/rock ratio is higher, or if isotopic equilibration is incomplete. This is consistent with estimates of the temperature at the ‘roots' of the Kerch mud volcanoes of 70–127 °C, based on Mg-Li and Na-Li geothermometers [7].

## 6. Conclusions

This first systematic study shows that several groups of groundwater with distinct stable isotopic compositions exist in Crimea.

Natural springs in the Main Range of the Crimean Mountains and the Crimean Piedmont yielded a restricted range of values (δ^18^O = −10.5 to −8.0 ‰, δ^2^H = −72 to −58 ‰). On average, these values are somewhat lower than the weighted mean values of precipitation recorded over 3–4 years of monitoring in the Crimean Mountains and the Piedmont (Chatyr-Dag and Simferopol stations); [20]. The isotopically depleted character of the spring water is explained by preferential recharge at higher elevations and during winter, when evaporation is low.

Water tapped by boreholes in Crimea can be separated into three groups. One group has isotopic properties similar to those of the springs. These borehole waters also have isotope values slightly more negative than the mean precipitation. The second group shows significantly depleted isotopic compositions (δ^18^O = −13.3 to −12.0 ‰, δ^2^H = −95 to −82 ‰). Elevated contents and complex compositions of dissolved gases, as well as elevated temperatures suggest the admixture of deep-seated groundwater at some of these sites. The very low isotope values argue for recharge during colder Pleistocene times. The third group of boreholes taps waters of high isotope values (δ^18^O = −2.5 to +1.0 ‰, δ^2^H = −24 to −22 ‰). The oxygen isotope enrichment is disproportional, and the data points are shifted to the right of the Crimean LMWL. The boreholes are 1600−1800 m deep and discharge highly mineralized (25.3 g L^−1^) thermal (65°C at orifice) water. The origin of these waters is presently unknown, but the isotopic characteristics suggest water–rock exchange reactions.

Groundwater on the Kerch peninsula forms a separate group. Waters associated with mud volcanoes have distinct isotope characteristics (δ^18^O = −1.6 to +9.4 ‰, δ^2^H =−30 to −18 ‰). Restricted variability of δ^2^H along with strongly variable and generally high δ^18^O values indicates water–rock isotopic exchange at elevated temperatures (>95 °C), which is consistent with independent temperature estimates of the root zones of these mud volcanoes. Springs on the Kerch peninsula show variable isotopic compositions. One epikarstic spring has a composition consistent with meteoric recharge, while the others show high δ^18^O and δ^2^H values, indicating various degrees of mixing with isotopically heavy groundwater typical of mud volcanoes.

## Geolocation information

Coordinates of Crimea: 45.3453 ° N, 34.4997 ° E

## Supplementary Material

IEHS_Dublyansky_SUPPLEMENT_REV.pdf

## Data Availability

Data discussed in this paper are available at doi:10.17632/7bhp3v3wcs.1.

## References

[1] Dublyansky VN, Dublyanskaya GN. Karstovaya respublika (karst Kryma i ego problemy) [The karst republic (karst of Crimea and its issues)]. Simferopol; 1996 Russian.

[2] Gordievich VA, Kurishko VA, Lychagin GA. Gidrogeologiya Kryma i perspektivy ego neftegazonosnosti [Hydrogeology of Crimea and perspectives of its oil- and gas potential]. Kiev: AN USSR; 1963; Russian.

[3] Barabanov LN, Lychagin GA, Mesiats IA, et al., editors. Gidrogeologiya SSSR, tom VIII, Krym [Hydrogeology of USSR, vol. VIII, Crimea]. Moscow: Nedra; 1970 Russian.

[4] Kayukova EP, Yurovsky YG. Vodnije resursy Kryma [Water resources of Crimea]. Geoecol Eng Geol Hydrogeol Geocryol. 2016;1:25–32. Russian.

[5] Miran’kov DB. Problemi preodoleniya deficita vodnykh resursov v Respublike Krym: Adaptatsyia zarubezhnogo opyta [Problems of overcoming water resource deficit in the Republic of Crimea: Adaptation of foreign experience]. Scholarly Notes Crimean Federal University, Economics and Management. 2018;4(70):93–107. Russian.

[6] Aydarkozhina AS, Lavrushin VY. Usloviya formirovaniya giazevulkanicheskikh fluidov Krima, po dannim izmereniya δD, δ^18^O, δ^13^C, δ^15^N [Conditions of formation of mud volcano fluids from measurement of δD, δ^18^O, δ^13^C, δ^15^N]. In: Proceedings of the XXI Symposium on Isotope Geochemistry; 2016 Nov 15–17; Moscow. Moscow: Vernadsky Institute of Geochemistry, Russian Academy of Sciences, p. 135–138. Russian.

[7] Ershov VV, Levin BV. Novije dannije o veschestvennom sostave produktov dejatelnosti griazevikh vulkanov Kerchenskogo poluostrova [New data on material composition of the products of activity of mud volcanoes of the Kerch peninsula]. Dokl Akad Nauk. 2016;471(1):1–5. Russian.

[8] Seletsky YB, Pribluda VD, Poliakov VA, et al. Ispolzovanije kontsentratsij tiazhologo izotopa kisloroda pri izuchenii podzemnikh vod zakarstovannikh karbonatnikh massivov Gornogo Krima [Using concentrations of heavy isotope of oxygen in studies of ground waters in karstified carbonate massifs of the Crimean Mountains]. Water Res. 1982;4:83–90. Russian.

[9] Dublyansky YV, Klimchouk AB, Amelichev GN, et al. Izotopnyi sostav atmosfernikh osadkov i karstovikh istochnikov severo-zapadnogo sklona Krimskikh gor [Isotopic composition of atmospheric precipitation and karst springs of the northwest slope of the Crimean Mountains]. Speleol Karstol. 2012;9:14–21. Russian.

[10] Kayukova EP. Formirovanie izotopnogo sostava prirodnikh vod Gornogo Kryma pod vlijaniem estestvennikh processov [Formation of isotopic composition of natural waters of the Crimean Mountains under the influence of natural processes]. Vestn St Peterbg Univ. 2016;7(2):11–26. Russian.

[11] Amelichev GN, Tokarev IV, Tokarev SV, et al. Kompleksnaya otsenka vozrasta i ustanovleniye usloviy formirovaniya minerl’nikh vod ‘Bishuli’ (Ravninniy Krim) na osnove izotopno-geokhimicheskikh dannykh [Comprehensive assessment of age and determination of conditions of formation of mineral water ‘Bishuli’ (Crimean Plains) on the basis of isotope geochemistry data]. Scholarly Notes Crimean Federal University, Geography, Geology. 2017;3(69):130–150. Russian.

[12] Yudin VV. Geodinamika Kryma [Geodynamics of Crimea]. Simferopol: DIP; 2011; Russian.

[13] Shestopalov VM, Blinov PV, Lyutyi GG, et al. Suchasni princypy gidrogeologichnogo rainuvannya [Modern principles of hydrogeological regionalization]. Zbirnik naukovyh prac UkrDGRI. 2010;3–4:147–157. Ukrainian.

[14] Gor’kova LG, Lymar NB. Karta jestestvennoy zashchischennosti podzemnyh vod Ukrainskoy SSR, Masshtab 1: 200 000, Krymskaja oblast’, Objasnitelnaja zapiska [Map of natural protection of underground waters of the Ukrainian SSR, Scale 1: 200 000, Crimean region, Explanatory notes]. Kiev: Mingeo USSR; 1987; Russian.

[15] Lushchik AV, Morozov VI, Meleshin VP, et al. Podzemnyye vody karstovykh platformennyh oblastey yuga Ukrainy [Groundwaters of platform karst regions of the south of Ukraine]. Kiev: Naukova dumka; 1981; Russian.

[16] Lushchik AV, Morozov VI, Pavkin VP, et al. Osobennosti formirovaniya podzemnyh vod v zapadnoy chasti Ravninnogo Kryma (na promere buhty Ocheretay) [Particularities of the groundwater formation in the western part of the Crimean Plains (on an example of Ocheretay cave)]. Geol J. 1985;45(3):101–107. Russian.

[17] Gehre M, Geilmann H, Richter J, et al. Continuous flow ^2^H/^1^H and ^18^O/^16^O analysis of water samples with dual inlet precision. Rapid Commun Mass Spectrom. 2004;18:2650–2660. doi: 10.1002/rcm.1672 15481101

[18] Spötl C, Fairchild IJ, Tooth AF. Speleothem deposition in a dynamically ventilated cave, Obir Caves (Austrian Alps). Evidence from modern cave air and drip water monitoring. Geochim Cosmochim Acta. 2005;69:2451–2468. doi: 10.1016/j.gca.2004.12.009

[19] van Geldern R, Barth JAC. Optimization of instrument setup and post-run corrections for oxygen and hydrogen stable isotope measurements of water by isotope ratio infrared spectroscopy (IRIS). Limnol Oceanogr Meth. 2012;10:1024–1036. doi: 10.4319/lom.2012.10.1024

[20] Dublyansky YV, Klimchouk AB, Tokarev SV, et al Stable isotopic composition of atmospheric precipitation on the Crimean Peninsula and its controlling factors. J Hydrol. 2018;565:61–73.

[21] Dublyansky VN, Vakhrushev BA, Amelichev GN, et al. Krasnaya peschera: Rezul’tati kompleksnikh issledovanij [Krasnaya cave. Results of the comprehensive karstological studies]. Moscow: RUDN; 2002; Russian.

[22] Klimchouk AB. Epikarst: gidrogeologiya, morfogenez i evolutsiya [Epikarst: hydrogeology, morphogenesis and evolution]. Simferopol: Sonat; 2009; Russian.

[23] Schwartz K, Barth JAC, Postigo-Rebollo C, et al. Mixing and transport of water in a karst catchment: a case study from precipitation via seepage to the spring. Hydrol Earth Syst Sci. 2009;13:285–292. doi: 10.5194/hess-13-285-2009

[24] Perrin J, Jeannin P, Zwahlen F. Epikarst storage in a karst aquifer: a conceptual model based on isotopic data, Milandre test site, Switzerland. J Hydrol. 2003;279:106–124. doi: 10.1016/S0022-1694(03)00171-9

[25] Dublyansky VN. Karstovije pescheri i shakhti Gornogo Krima [Karst caves and shafts of the Crimean Mountains]. Leninhrad: Nauka; 1977; Russian.

[26] Vakhrushev BA, Vakhrushev IB. Rol’ karstovikh kondensatcionnikh vod v vodnom khoziaystve antichnikh i srednevekovikh poseleniy Kerchenskogo poluostrova [The role of karst condensation water in the water management of antique and medieval settlements of Kerch peninsula]. Culture of the Nations in the Black Sea Region. 1999;10:7–10. Russian.

[27] Clark ID, Fritz P. Environmental isotopes in hydrogeology. Boca Raton (FL): CRC Press; 1977.

[28] Shniukov EF, Gnatenko GI, Nesterovsky VA, et al. Griazevoy vulkanizm Kerchensko-Tamanskogo regiona [Mud volcanism of the Kerch-Taman Region]. Kiev: Naukova dumka; 1992; Russian.

[29] Kurishko VA, Mesiats IA, Terdovidov AS. Gidrogeologija griazevogo vulkanizma Kercheskogo poluostrova [Hydrogeology of mud volcanism of the Kerch peninsula]. Geol J. 1968;28(1):49–59. Russian.

[30] Buyakaite MI, Lavrushin VY, Pokrovsky BG, et al. Izotopniye sistemi strontsiya i kisloroda v vodakh griazevikh vulkanov Tamanskogo poluostrova [Isotope systems of strontium and oxygen in waters of mud volcanoes of the Taman’ peninsula]. Lith Min Resour. 2014;1:52–59.

