# Supplementary material for: Groundwater of the Crimean peninsula: a first systematic study using stable isotopes
Source: Isotopes Environ Health Stud. 2019 Aug 16;55(5):419–37. doi: 10.1080/10256016.2019.1650743 (PMC6816486; doi:10.1080/10256016.2019.1650743)
Supplement: IEHS_Dublyansky_SUPPLEMENT_REV.pdf [file GIEH_A_1650743_SM4314.pdf]

depositphotos.com / Mondi.h

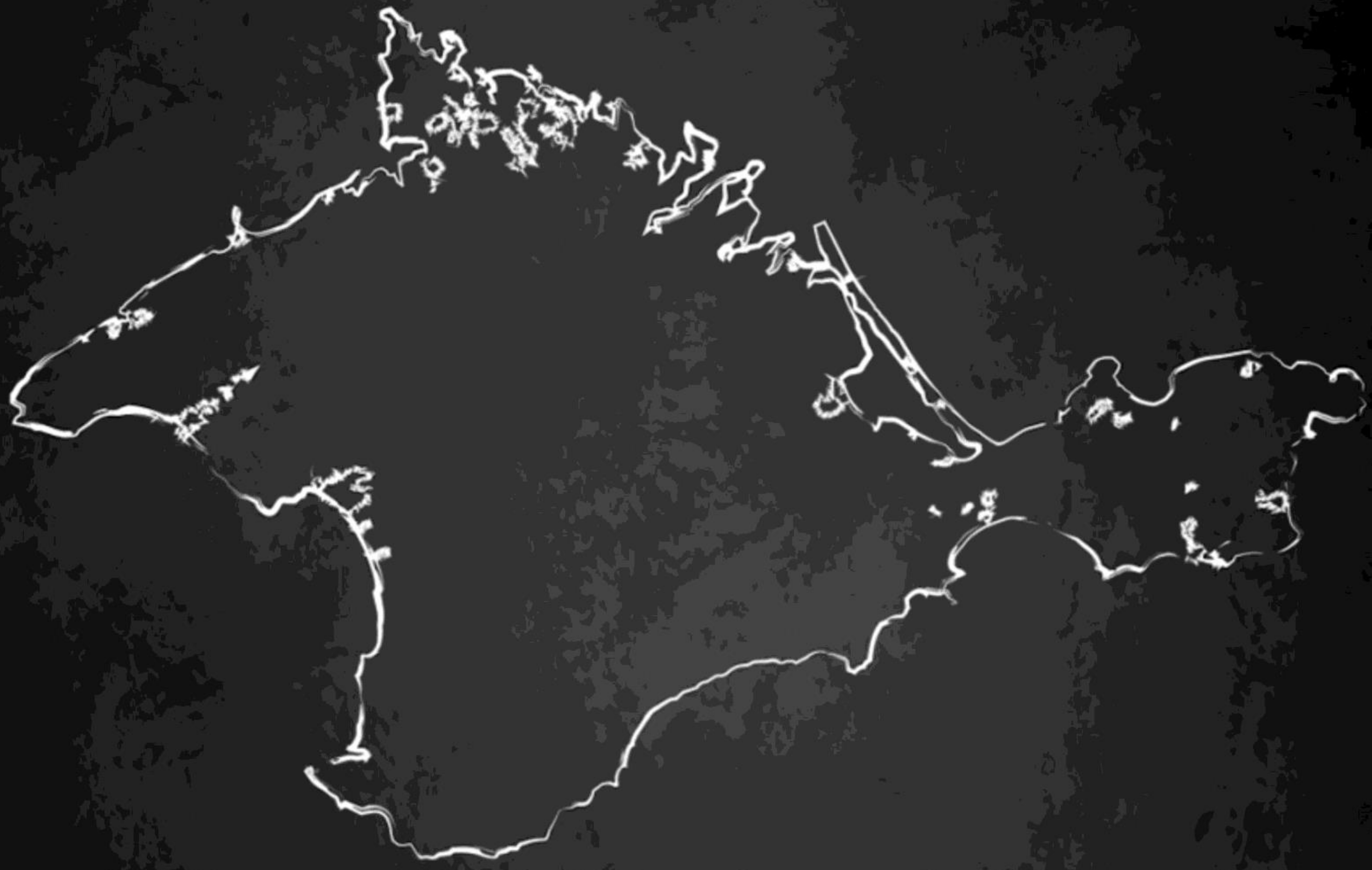

Geology and hydrogeology of Crimea

Yuri V. Dublyansky
Alexander B. Klimchouk
Sergey V. Tokarev
Gennady N. Amelichev
Christoph Spötl

2019

Supplemental materials to:
Groundwater of the Crimean peninsula: A first systematic study using stable isotopes.
Isotopes in Environmental & Health Studies

Geology and hydrogeology of Crimea

Supplemental materials to:
Groundwater of the Crimean peninsula: A first systematic study using stable isotopes.
Isotopes in Environmental & Health Studies.

Yuri V. Dublyansky[a], Alexander B. Klimchouk[b], Sergey V. Tokarev[c], Gennady N. Amelichev[c], and Christoph Spötl[a]

[a] *Institute of Geology, Innsbruck University, Innsbruck, 6020, Austria*
[b] *Institute of Geological Sciences, National Academy of Science of Ukraine, Kiev, 01054, Ukraine*
[c] *V. I. Vernadsky Crimean Federal University, Simferopol, 295007, Republic of Crimea*

Geology and hydrogeology of the Crimean peninsula have been researched extensively over the last two centuries. Yet, most of the results are only available in Russian and Ukrainian languages and commonly appear in publications that are presently out of print. In this document, we provide a brief summary of geology of Crimea, as well as a somewhat more detailed description of its hydrogeological features.

The Crimean peninsula comprises two main physiographic provinces, the Crimean Plains in the northern part and the Crimean Mountains in the south (Fig. 1). The Crimean Mountains comprise (from south to north) the Main, the Inner, and the Outer Ranges; the latter two are also known as the Crimean Piedmont (Predgorje).

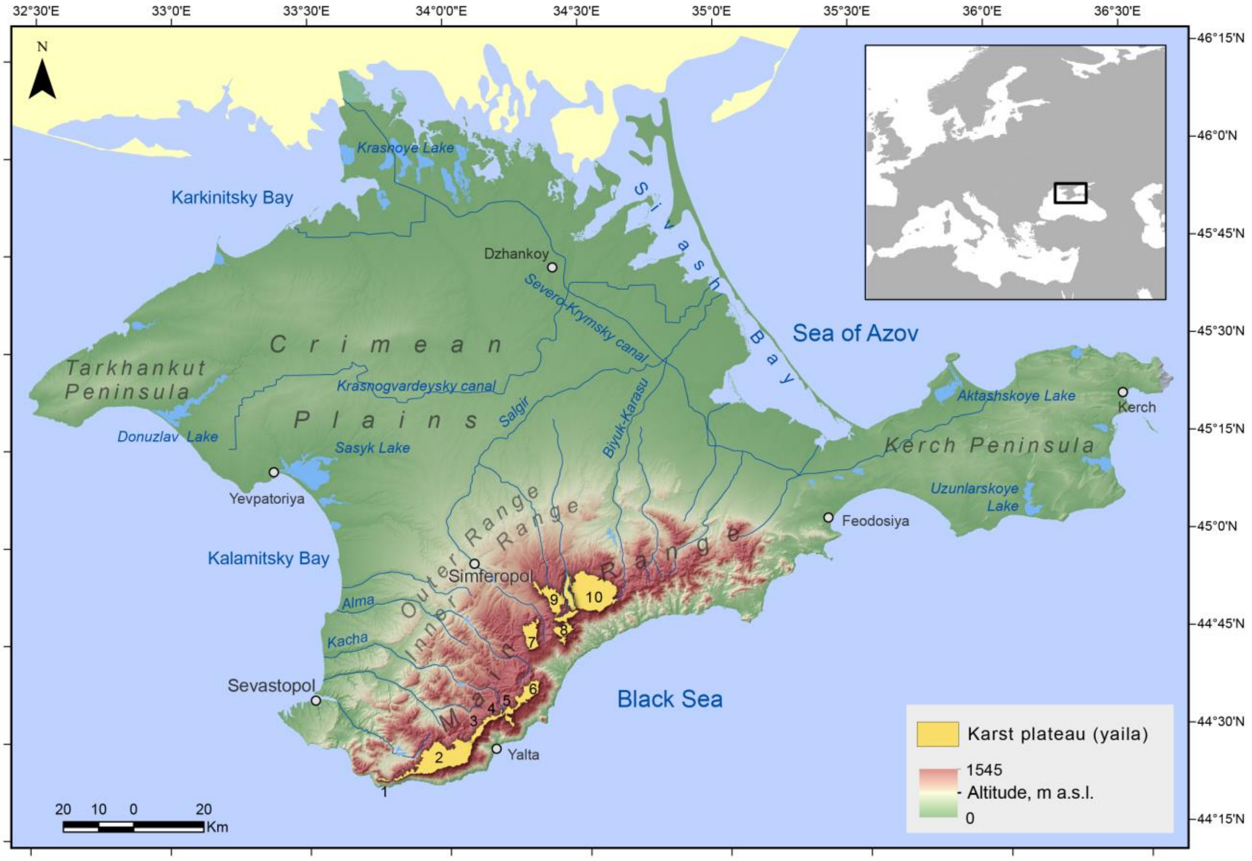

Fig. 1. Physiography of Crimea. Karst plateaus (yaylas): 1 – Baydarskaya; 2 – Ay-Petrinskaya; 3 – Yaltinskaya; 4 – Nikitskaya; 5 – Gurzufskaya; 6 – Babugan; 7 – Chatyr-Dag; 8 – Demerdzhi; 9 – Dolgorukovskaya; 10 – Karabi.

Geology

The physiographic provinces of the Crimean Plains and the Crimean Mountains correspond to two regional tectonic structures: the epi-Hercynian Scythian Plate and the Alpine Crimean Mountains fold-and-thrust structure (Fig. 2). These structures are separated by a regional deep-seated fault, which has also been interpreted as a collision suture (Piedmont Suture; Yudin, 2011). This low-angle north-dipping thrust fault was active from the early Jurassic until the early Cretaceous. The lower tectonostratigraphic story in the Crimean Mountains is composed mainly of Triassic shale-dominated flysch deposits and Upper Jurassic and Lower Cretaceous limestones. The limestones form rigid massifs interpreted as olistoliths (Yudin, 2011). The upper tectonostratigraphic story comprises alternating shales/marls and carbonate rocks of Upper Cretaceous, Paleogene, and Neogene age, which were moderately deformed during re-activation of the suture in Cenozoic times and form a homocline dipping 5°–15° to the northwest and north.

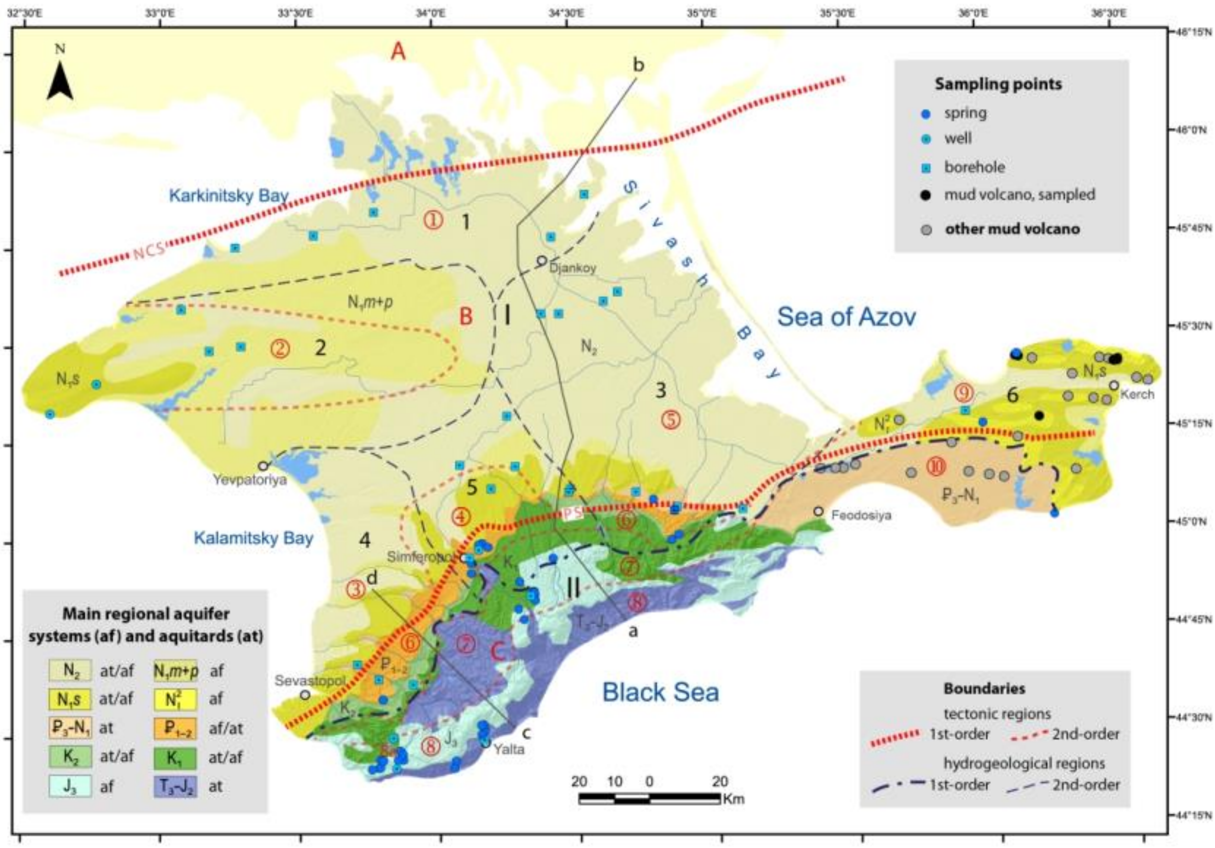

Fig. 2. Tectonic and hydrogeological overview map of Crimea. Tectonic regions (capitalized letters; after Yudin, 2011): 1st-order regions: A - Ukrainian Plate, B - Scythian Plate, C - Crimean Mountains fold-thrust region. Boundaries between 1st-order regions: NCS - North-Crimean suture, PS - Piedmont suture. 2nd-order regions (red circled numerals): 1 - Karkinitsky depression, 2 - Tarkhankutsky-Novoselovsky uplift, 3 - Alminskaya depression, 4 - Simferopol uplift, 5 - Indolo-Kubansky depression, 6 - Cuesta homocline, 7 - Piedmont structural zone, 8 - Mountain structural zone, 8a – Baydarsky depression, 9 - North-Kerch retrothrust zone, 10 - South-Kerch thrust zone. Hydrogeological regions: 1st-order regions (Roman numerals; after Lushchik et al., 1981): I - Prichernomorsky groundwater system (artesian basin), II - Crimean Mountains groundwater system. 2nd order regions in the Crimean Plains and the Kerch peninsula (numerals; after Lushchik et al., 1981): 1 - NorthSivash, 2 - Novoselovsky; 3 - Belogorsky; 4 - Alminsky; 5 - Simferopolsky, 6 - Kerch system of small artesian basins. Lines a-b and c-d correspond to hydrogeological cross-sections shown in Fig. 3.

From the Late Cretaceous to the Paleogene, the northern periphery of the fold-and-thrust belt of the Crimean Mountains was buried by marine sediments, which become more abundant further to the north in the Scythian Plate (the Crimean Plains). The Upper Jurassic limestone massifs that now form most of the Main Range were stripped off their Cretaceous-Paleogene cover during the Neogene-Quaternary uplift. Ensuing erosion separated the Main and the Inner Ranges. The geological and hydraulic continuity of the homocline was completely breached in the northwestern sectors, but was preserved in the central and eastern parts of the mountain region.

The Inner and the Outer ranges, constituting the Crimean Piedmont, show a cuesta morphology and rim the Crimean Mountains from the northwest and north, forming a 130 km-long arch. Cuestas of the Inner Range are more prominent and are built up of Upper Cretaceous, Paleocene and Eocene carbonate strata. Further to the northwest and north, Cretaceous to Paleocene strata show a steeper dip and plunge to great depth. Cuestas of the Outer Range are built up of Neogene carbonates overlying Paleogene or Cretaceous strata. The thickness of the Neogene strata increases northward, reaching several hundred meters in the subsurface of the Crimean Plains.

The basement of the Scythian Plate is composed of strongly deformed Paleozoic rocks dominated by metamorphosed shales and carbonates. The main tectonic structures within the Crimean Plains are the Karkinitsky depression in the north, the Indolo-Kubansky depression in the east, the Simferopolsky uplift in the center, the Tarkhankut-Novoselovskiy uplift in the west, and the Alminskaya depression in the southwest.

Hydrogeology

Two main hydrogeological domains are distinguished within the Crimean peninsula (Fig. 2), the Crimean Mountains groundwater system and the southern part of the Prichernomorsky groundwater system (artesian basin) which encompasses the Crimean Plains and the Piedmont (Shestopalov et al., 2010). Structural depressions in the basement of the Scythian Plate within the Crimean part of the Prichernomorsky system form second-order artesian basins (North-Sivash, Belogorsky and Alminsky). The Kerch peninsula hosts a suite of small-scale artesian basins. Schematic hydrogeological cross-sections are presented in Fig. 3.

Crimean Mountains groundwater system

The main groundwater resources in the Crimean Mountains are associated with the karstified Upper Jurassic limestones, which reach a thickness of up to 1 km. Terrigenous and terrigenous-volcanogenic deposits form the basement of the karstified plateaus in the Main Range (yailas) and show low hydraulic conductivities. The plateaus serve as the main recharge area and possess a mature epikarst. Limestone massifs of the Main Range provide relatively small storage of karst water in the phreatic zone, which discharges via more than 2000 springs located on the periphery of the plateaus. More than half of these springs are located within the

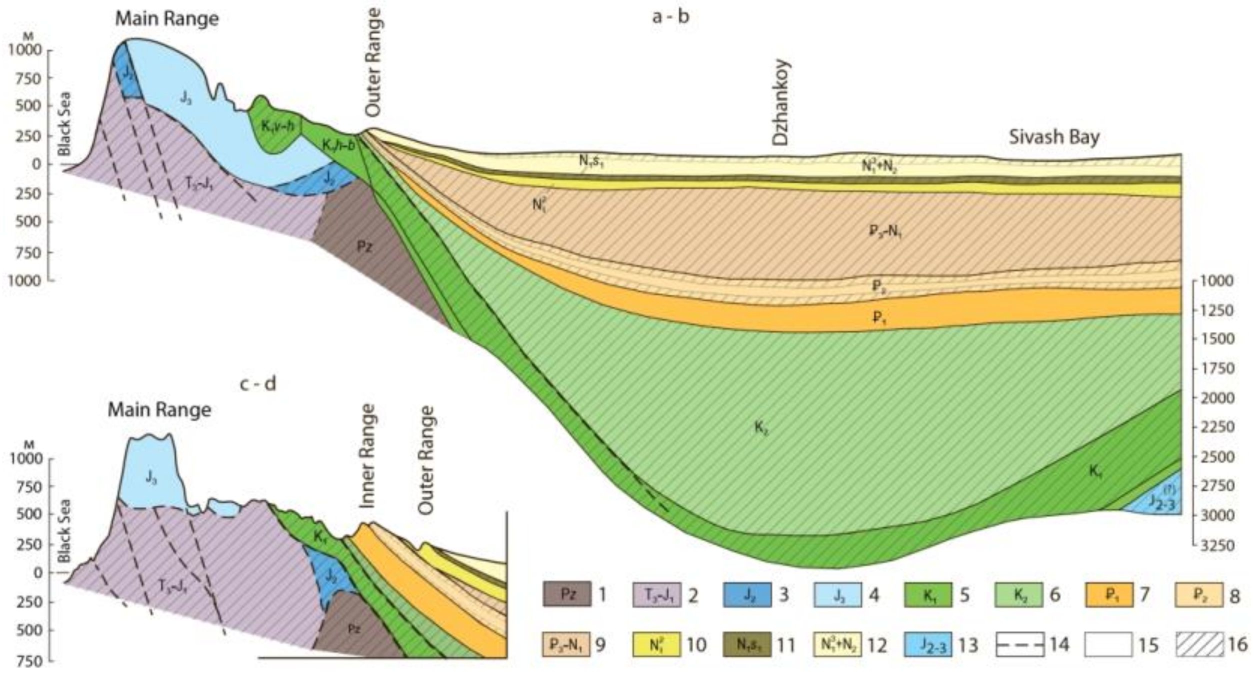

Fig. 3. Simplified hydrogeological cross-sections, approximately corresponding to lines a-b and c-d in Fig. 2. Modified from Barabanov et al. (1970). 1 - Paleozoic metamorphosed rocks (aquitards); 2 - Triassic / Lower Jurassic shales of the Tavricheskaya series (aquitards); 3 - Middle Jurassic sandstones, shales and conglomerates (aquitards); 4 - Upper Jurassic limestones (karst aquifers); 5 - Lower Cretaceous sandstones, conglomerates and limestones (aquitards, locally aquifers); 6 - Upper Cretaceous limestones and marls (locally fractured/karst aquifers), with marls and sandstones at the base (aquitards); 7 - Paleocene limestones and marls (fractured/karst aquifers); 8 - Eocene limestones and marls (fractured/karst aquifers), with shales at the base (aquitards); 9 - Oligocene clays of the Maikop series (aquitards); 10 - Middle Miocene sands and limestones (aquifers); 11 - Lower-Middle Sarmatian clays (aquitards); 12 - Upper Miocene – Pliocene limestones and sands (aquifers), with clays in upper part (aquitards); 13 - sandy mudstones, presumably of the Middle to Upper Jurassic (locally fractured aquifers); 14 - fault lines; 15 – predominantly aquifers formations; 16 - predominantly aquitards formations. Vertical exaggeration ca. 17x.

200-600 m a.s.l. altitudinal interval. Discharge of the karst springs ranges from a few to hundreds of liters per second. Springs exceeding 10 L s^{-1} comprise only 4 % of the springs. Their cumulative discharge, however, is 83 % of the total spring discharge (Barabanov et al., 1970). Out of those, 19 springs with a discharge exceeding 100 L s^{-1} account for ca. 75 % of the total discharge. These numbers indicate that the groundwater flow is focused into a few highly transmissive karst conduits.

Where Upper Jurassic limestones plunge underneath the Cretaceous cover outside the massifs (e.g., western periphery of the Ay-Petrinskaya yaila and between the Dolgorukovskaya and Chatyr-Dag yailas in the central sector, northern periphery of the Dolgorukovskaya yaila and the northern and eastern periphery of the Karabi yaila in the eastern sector) they form confined aquifers providing additional recharge for the adjacent Prichernomorsky system. For

example, to the west of Ai-Petri, groundwater from the massif flows towards the Baydarsky depression (a small artesian basin) after which part of the water discharges as submarine springs near cape Aya in the southwest, while another part feeds the Alminsky second-order artesian basin in the north-west (Barabanov et al., 1970). Waters recharging in the Dolgorukovskaya and Karabi yailas feed the aquifers of the Prichernomorsky system in the Simferopol uplift and the Belogorsky basin (Barabanov et al., 1970, Gor'kova, Lymar, 1987).

Groundwater in the Upper Jurassic limestones, even when confined (e.g., in the Baydarsky depression), belongs to the zone of active water exchange and shows 0.3 to 0.5 g L^{-1} total dissolved solids (TDS). Some boreholes located outside the massifs at intersections of tectonic faults tap confined Na-Cl-type (e.g., Krasnaya cave area) or Na-HCO_3-type (e.g., Chernye Vody area) waters exceeding 3 g L^{-1}. These waters are commonly enriched in He, Rn, H_2S, CH_4, and higher hydrocarbons, suggesting admixture of deep-seated fluids. High TDS, high contents of dissolved gases (CO_2 and H_2S) and minor elements were also reported from some springs in the eastern part of the Crimea Mountains associated with faults (Barabanov et al., 1970, Gor'kova, Lymar, 1987, Lushchik et al., 1981).

Southern part of the Prichernomorsky system

The Prichernomorsky system extends as far south as the Crimean Piedmont, where Upper Cretaceous, Paleogene and Neogene strata are uplifted, tilted toward north-northwest and exposed within the Inner and the Outer Ranges and on the northern slope of the Crimean Mountains. This is the recharge area of the groundwater system located underneath the Crimean Plains.

The upper aquifers of the system are unconfined and the rocks crop out in the Southern Longitudinal depression in the Piedmont as well as in the Tarkhankut and the Novoselovsky uplifts. The aquifers are recharged by infiltration of meteoric precipitation and, partly, by sub-river-channel flow on the northern slope of the Main Range and in the Piedmont, where valleys incise into Cretaceous, Paleogene and Neogene strata. As the groundwater flows further (and deeper) towards the Crimean Plains it acquires significant hydraulic head. The pressure difference between the recharge area on the Piedmont and the submerged areas may exceed 30 bar in the Neogene aquifers and 100 bar in Upper Cretaceous aquifers (Barabanov et al., 1970, Lushchik et al., 1981). The Aptian-Albian and some Upper Cretaceous strata, the thick

Oligocene-Lower Miocene Maikop series deposits, as well as thin but continuous beds of Lower Sarmatian clays act as aquitards. The description of aquifers that follows is based on reports: Barabanov et al. (1970), Gor'kova, Lymar (1987), Lushchik et al. (1981), Lushchik et al. (1987).

Neogene aquifers

The Neogene sequence in the Crimean Plains hosts three aquifers: Middle to Upper Pliocene sands and gravels, carbonates of Sarmatian to Pontian age, and undifferentiated Middle Miocene carbonates. The upper aquifer is only 10 to 40 m thick, separated into distinct horizons by shale aquitards, and confined by the overlying Pleistocene and Pliocene shales. The aquifer is recharged primarily by leakage from the underlying confined aquifer. The Sarmatian-Pontian aquifer is subdivided by internal aquitards and is underlain by Lower Sarmatian clays forming a regional aquiclude. The Middle Miocene aquifer comprises a layer of limestone and underlying sands. The transmissivity of the Neogene deposits is highly variable (Lushchik et al., 1981). The Pontian beds have the highest transmissivity reaching 2.3×10^{-1} m^2 s^{-1}. The hydraulic conductivity of this horizon may be as high as 6.4×10^{-7} m s^{-1}. The transmissivity of the Sarmatian and Maeotian horizons varies between 3.5×10^{-3} and 1.4×10^{-2} m^2 s^{-1}, and that of the Middle Miocene horizon from 1.2×10^{-4} to 5.8×10^{-4} m^2 s^{-1} (Lushchik et al., 1981).

The Crimean Piedmont forms the outer recharge area of the Neogene aquifers and the groundwater flows north- and northeastward. Approximately at the latitude of the town of Dzhankoy the flow splits; one branch continues northward and another one is deflected to the west, flowing north of the Tarkhankut uplift (the latter represents an additional recharge area). In the North-Sivash region, near the axial part of the Karkinitsky depression, this southerly flow meets the flow of freshwater from the north which recharged in the northern (continental) part of the Prichernomorsky system (Lushchik et al., 1981).

Hydraulic heads in the Middle Miocene aquifer typically exceed those in the Sarmatian-Pontian aquifer by 10 to 15 m, whereas heads in the latter exceed those in the Pleistocene aquifer by up to 10 m. This leads to upward leakage of water between the aquifers, documented along some faults as local hydrodynamic anomalies marked by natural gas emissions (Lushchik et al., 1981; 1987).

Paleogene aquifers

The Paleogene deposits host aquifers in the Middle Eocene and the Lower Paleocene, dominated by calcareous rocks. The aquifers are recharged in the Crimean Piedmont via infiltration of meteoric precipitation and sub-river channel flow in streams. Additional inflows from adjacent stratigraphic units are related to cross-formational flow along tectonized and often karstified zones. In the Piedmont, the waters are unconfined down to a depth of 50 m. Discharges of natural springs and wells typically do not exceed 2 L s^{-1}. The waters are of the Ca-HCO_3- and Mg-Na-HCO_3-type, containing up to 0.7 g L^{-1} TDS. As the aquifer rocks plunge northward toward the Crimean Plains, the Paleogene aquifers become confined, the groundwater acquires a substantial head, and TDS increase.

The Lower Paleocene aquifer is present on the southern sides of the Alminsky and the Belogorsky basins. It is confined by low-permeability marls and clays of Upper Cretaceous age below and by dense marls of the Kacha unit (Upper Paleocene) and Lower Eocene clays above. Toward the centers of the basins the thickness of aquifers increases from 45 to 75 m and the head reaches 1000–1500 m. Discharge is highly heterogeneous, drastically increasing in tectonized and karstified zones. In the recharge area waters are of the Ca-HCO_3- and Mg-Na-Ca-HCO_3-type and TDS reaches up to 0.7 g L^{-1}. With increasing depth the composition changes to Na-HCO_3-Cl- and Na-Cl-types, and the mineralization increases up to 18 g L^{-1}. These waters also contain minor components of Br and I and dissolved gases (mostly CH_4, as well as N_2, noble gases, and small amounts of O_2 and CO_2).

The Middle Eocene aquifer has a thickness of 20–40 m in the recharge area, increasing to 200–220 m in the submerged parts of the basins. It is mostly continuous in the southeastern side of the Alminsky basin and in the Simferopol uplift, and in the southern side of the North-Sivash depression. The water has a Ca-HCO_3-type composition and TDS values of 0.4–0.6 g L^{-1} in the recharge areas. Its composition evolves via a Ca-Na-HCO_3- and a Na-HCO_3-type into a Na-Cl-type, with up to 25 g L^{-1} TDS.

The two Paleogene aquifers are separated by marls and clays. This aquitard is discontinuous and is breached along fault zones. In areas where the aquitard is entirely absent the two aquifers merge. In the eastern sector, several kilometers to the north of the recharge area, there is a line of upwelling springs (Krivtsovo-Sennoe line), representing a marginal zone

of discharge of this plunging Paleogene aquifer. Discharge diminishes to the north of this line, suggesting lower conductivities in the deeper submerged parts of the aquifer.

Cretaceous aquifers

The Upper Cretaceous aquifer has its outer recharge area in the depression that separates the Main Range from the Piedmont and stretches along the foot of the Inner Range (the Southern Longitudinal depression). This aquifer is comprised of marls and marly limestone and hosts water of the Ca-Cl-SO_4–type with 1.7–3.2 g L^{-1} TDS. Toward north and northwest, the aquifer becomes confined and its composition changes to a Na-Cl-type with TDS values of 10–40 g L^{-1}.

The Lower Cretaceous aquifer comprises layers of sandstones, sands, Hauterivian and Barremian conglomerates and fractured limestones, as well as fractured sandstones and conglomerates within Albian shales. These rocks crop out on the northern slopes of the Main Range and, locally, in the Southern Longitudinal depression. The outcrops represent the outer recharge area for this aquifer. The thickness of the aquifer ranges from several meters to >100 m. The aquifer rocks plunge in northward direction down to a depth of 1500–2500 m (underneath the Novoselovsky uplift and the North-Sivash region). In these areas heads exceed 80–120 m above land surface (i.e., total head in the aquifer of 200–250 bar).

The water in the recharge area has a Ca-HCO_3 compositions and 0.3–0.6 g L^{-1} TDS. In the submerged parts, in the North-Sivash region, waters show a Na-Cl composition and 10–68 g L^{-1} TDS. The waters are also enriched in minor elements (Br and I). In the Novoselovsky uplift water contains CH_4 (up to 56 vol. %) and N_2 (up to 40 vol. %), as well as elevated amounts of He (Lushchik et al., 1987). CH_4-N_2-bearing waters are known from the Belogorsk area, and groundwater containing CH_4 and CH_4-N_2-CO_2 occurs in the northern and northwestern part of Sivash, respectively. In the western part of the Alminsky basin, in the Novoselovsky uplift, temperatures in the aquifers reach 50–58°C and in the northern Sivash region even about 100°C.

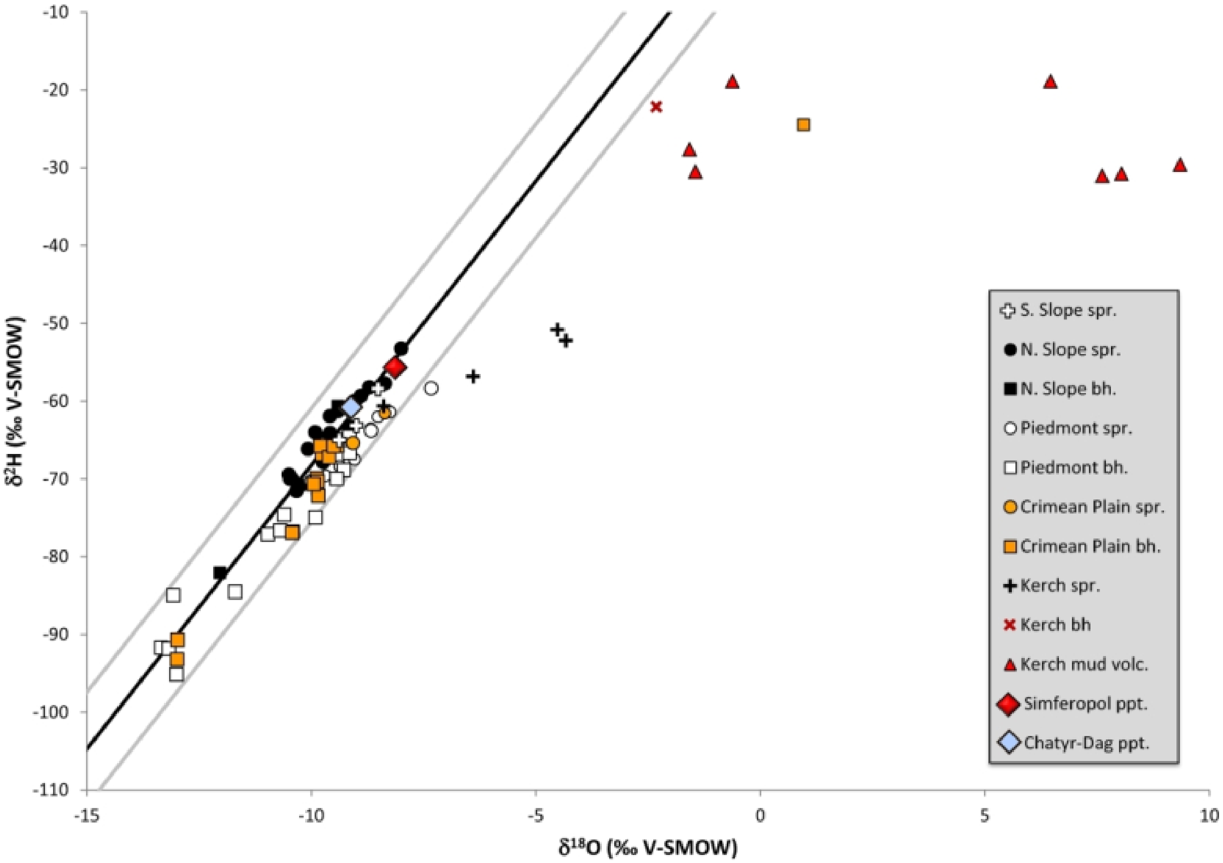

Fig. 4. Stable isotope properties of groundwater in Crimea. Data are plotted with respect to the Crimean LMWL (black line; Dublyansky et al., 2018) and the ±1‰ δ^{18}O band (grey lines). Large diamonds are weighted mean values of meteoric precipitation at two sites, Simferopol (290 m a.s.l.) and Chatyr-Dag (980 m a.s.l.), sampled in 2009–2011 (Dublyansky et al., 2018).

Stable isotope characteristics of groundwater in Crimea

Summary of the stable isotope analyses of the Crimean groundwater, discussed in the paper which is supported by this document ("Groundwater of the Crimean peninsula: A first systematic study using stable isotopes" Dublyansky et al., 2019) is shown in Fig. 4. The complete dataset is available at doi:10.17632/7bhp3v3wcs.1.

Acknowledgments

The study was supported by FWF grant P25716-N19 (YD) and RFBR–Council of Ministers of the Crimean Republic grants 16-45-910579r_a and 19-45-910008 (ST, GA). We thank A.V. Lushchik for sharing his insights on various aspects of the hydrogeology of Crimea.

References

Barabanov LN, Lychagin GA, Mesiats IA, et al. (eds.) (1970): Gidrogeologiya SSSR, tom VIII, Krym [Hydrogeology of USSR, vol. VIII, Crimea]. Moscow: Nedra. (in Russian).

Dublyansky YV, Klimchouk AB, Tokarev SV, Amelichev GN, Spötl C (2018): Stable isotopic composition of atmospheric precipitation on the Crimean Peninsula and its controlling factors. J Hydrol. 565:61-73.

Dublyansky YV, Klimchouk AB, Tokarev SV, Amelichev GN, Spötl C (2019): Groundwater of the Crimean peninsula: A first systematic study using stable isotopes. Isotopes in Environmental & Health Studies. (in press).

Gor'kova LG, Lymar NB. (1987): Karta jestestvennoy zashchischennosti podzemnyh vod Ukrainskoy SSR, Masshtab 1 : 200 000, Krymskaja oblast', Objasnitelnaja zapiska [Map of natural protection of underground waters of the Ukrainian SSR, Scale 1 : 200 000, Crimean region, Explanatory notes]. Kiev: Mingeo USSR. (in Russian).

Lushchik AV, Morozov VI, Meleshin VP, et al. (1981): Podzemnyye vody karstovykh platformennyh oblastey yuga Ukrainy [Groundwaters of platform karst regions of the south of Ukraine] Kiev: Naukova dumka. (in Russian).

Lushchik AV, Morozov VI, Pavkin VP, et al. (1985): Osobennosti formirovaniya podzemnyh vod v zapadnoy chasti Ravninnogo Kryma (na promere buhty Ocheretay) [Particularities of the groundwater formation in the western part of the Crimean Plains (on an example of Ocheretay cove)]. Geol J, 45(3):101–107. (in Russian).

Shestopalov VM, Blinov PV, Lyutyi GG, et al. (2010): Suchasni princypy gidrogeologichnogo rainuvannya [Modern principles of hydrogeological regionalization]. In: Zbirnik naukovyh prac UkrDGRI, 3–4:147–157. (in Ukrainian).

Yudin VV (2011): Geodinamika Kryma [Geodynamics of Crimea]. Simferopol: DIP. (in Russian).
